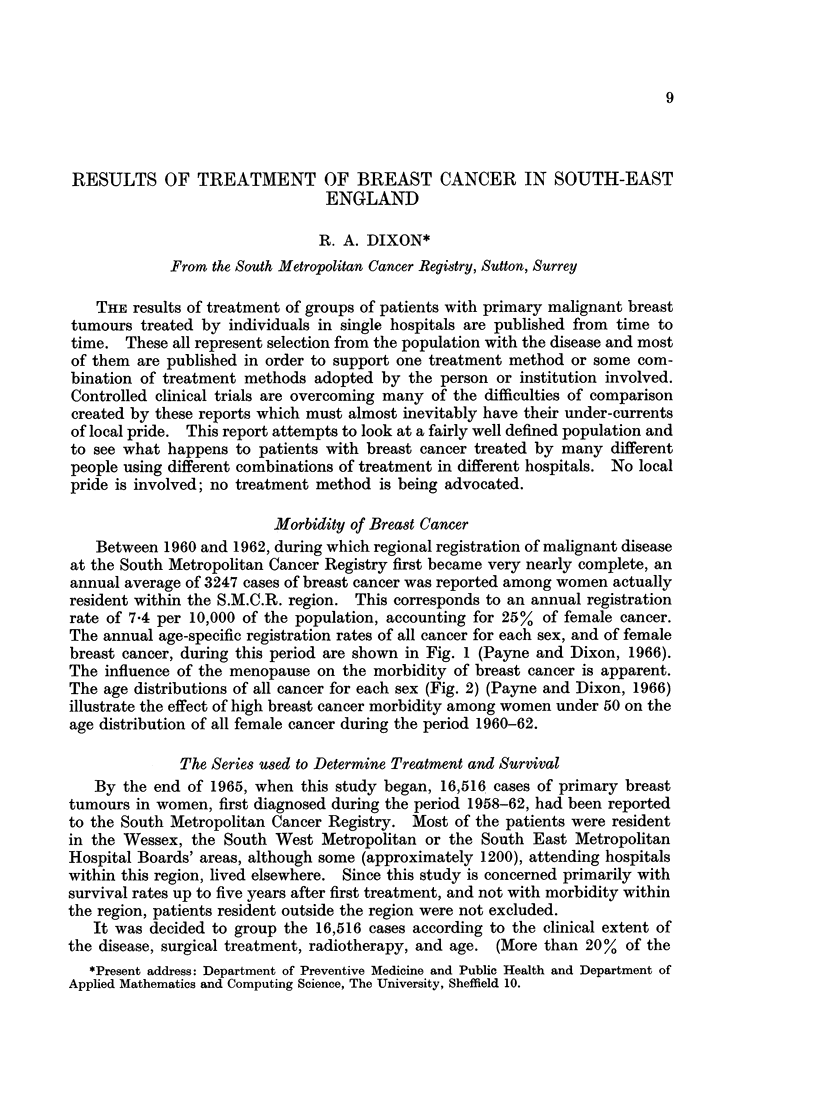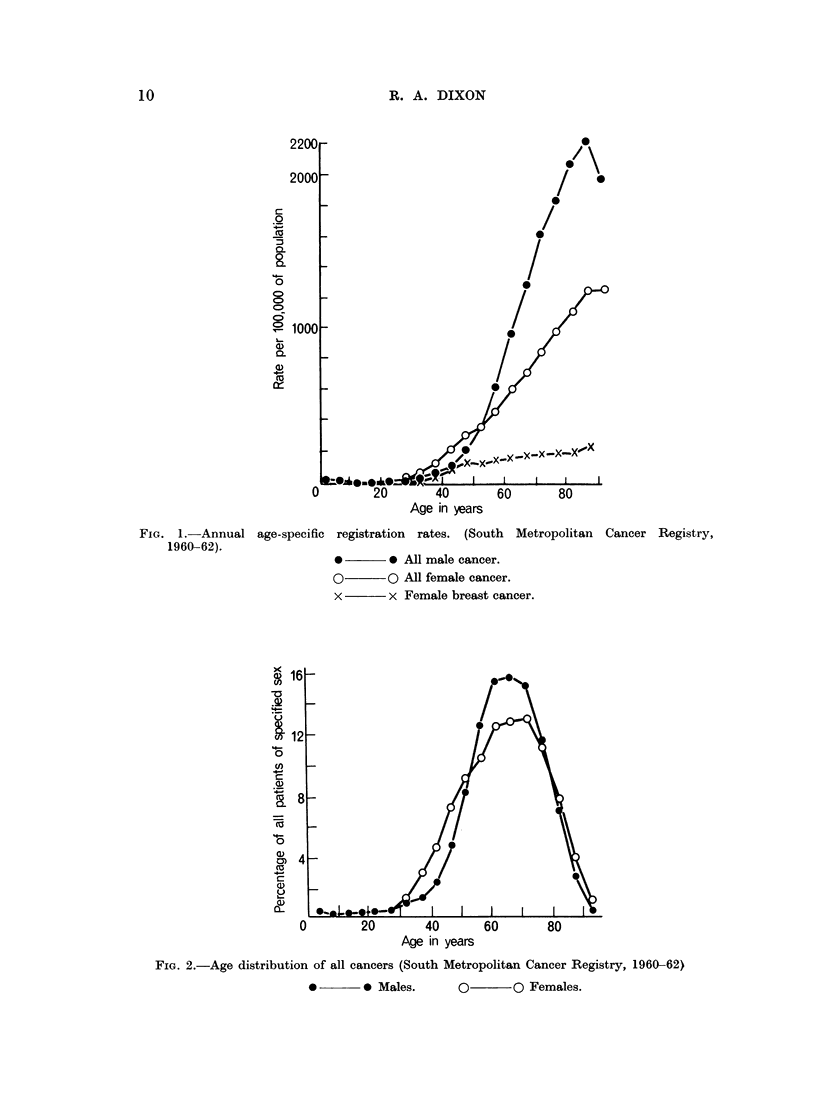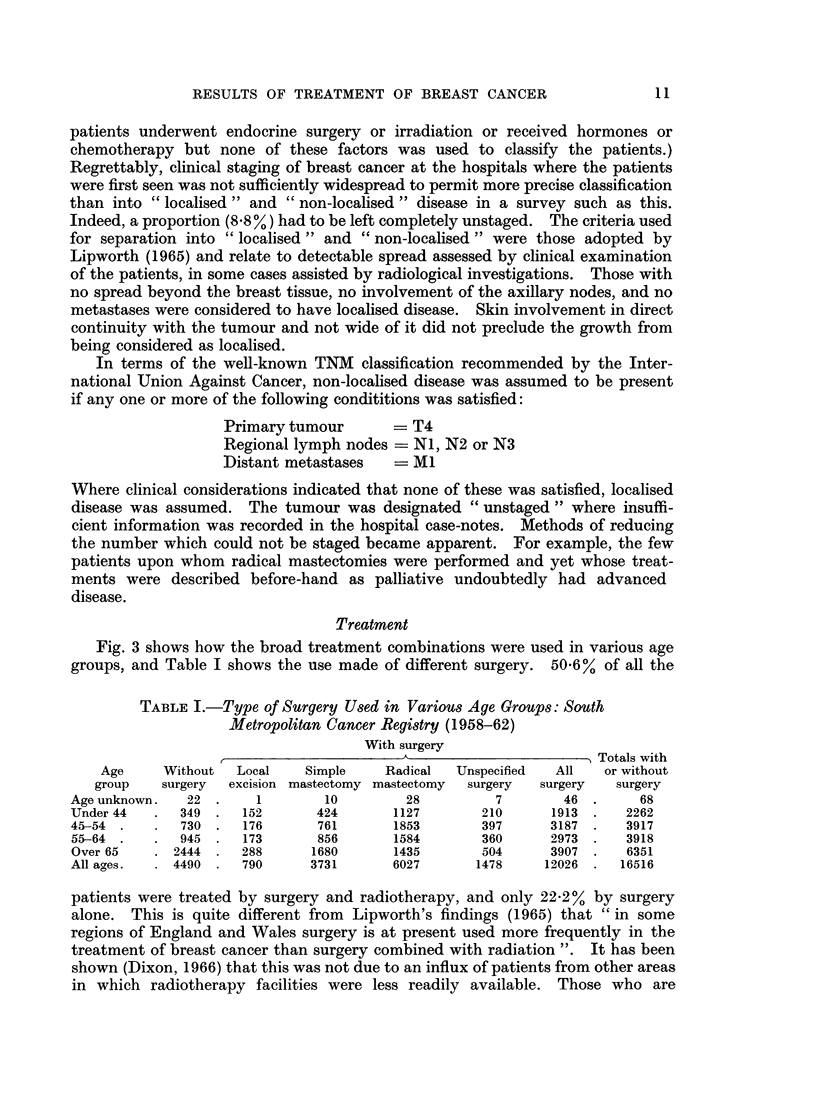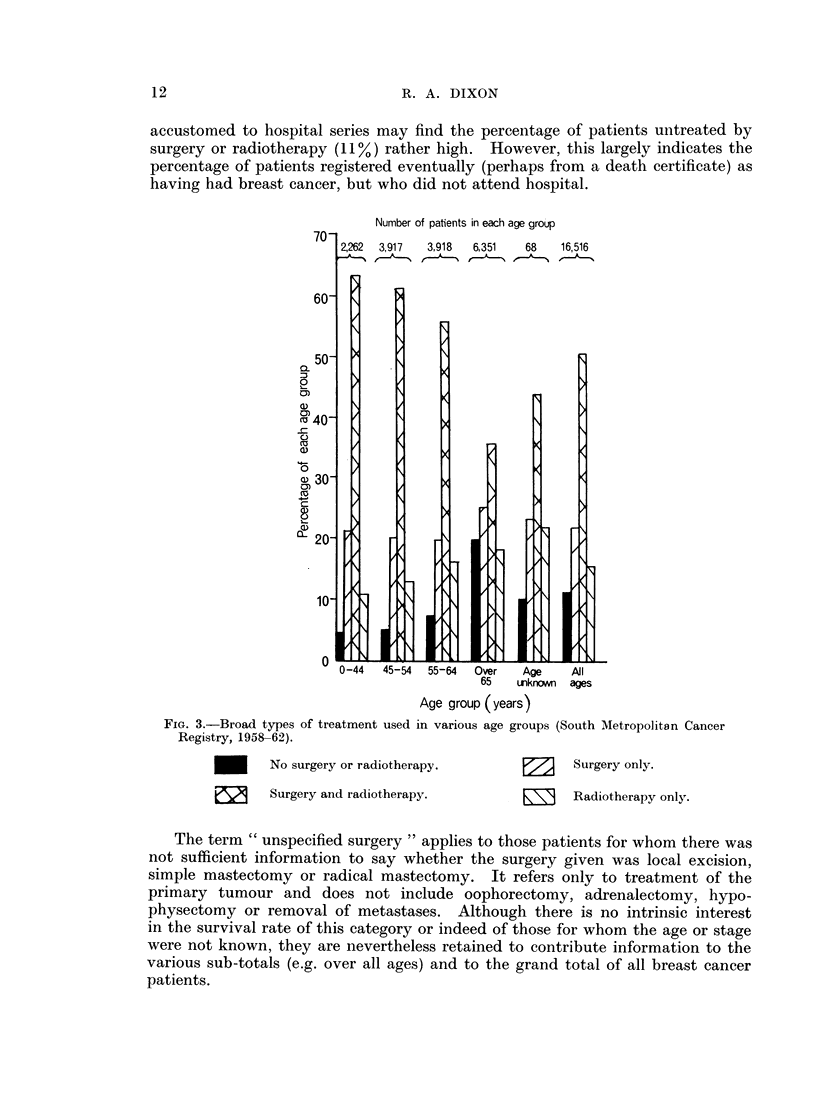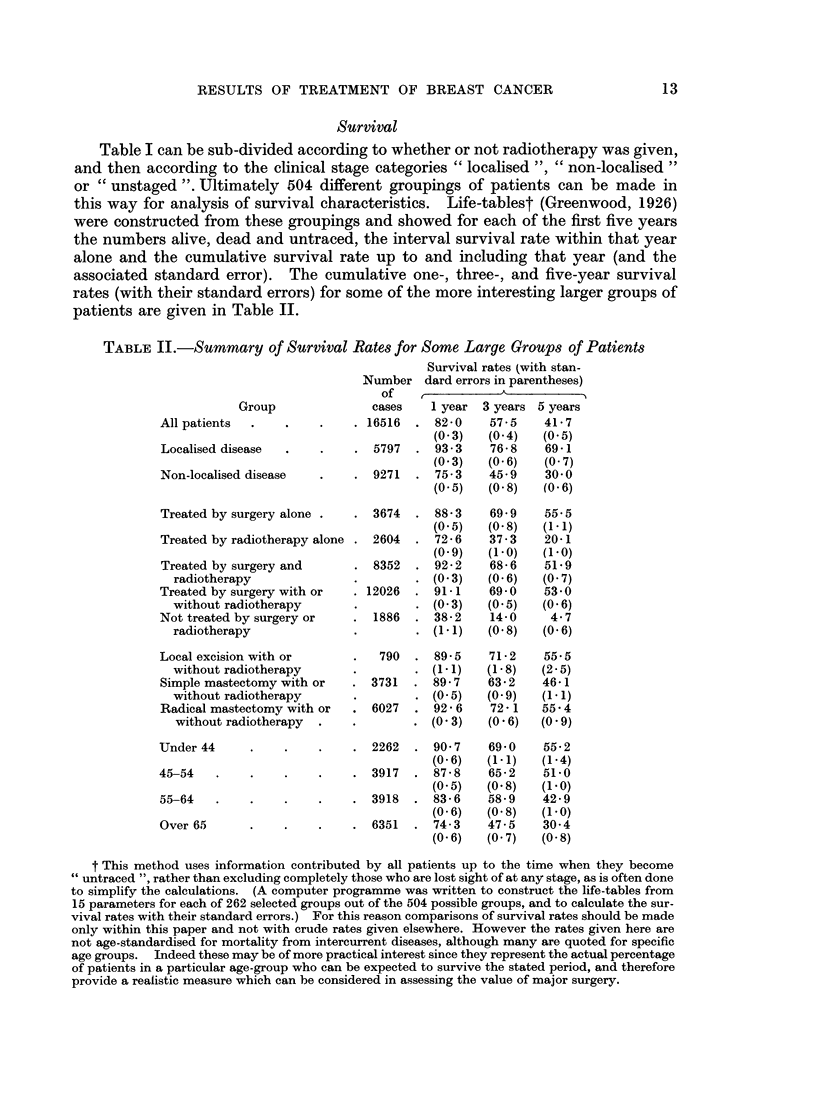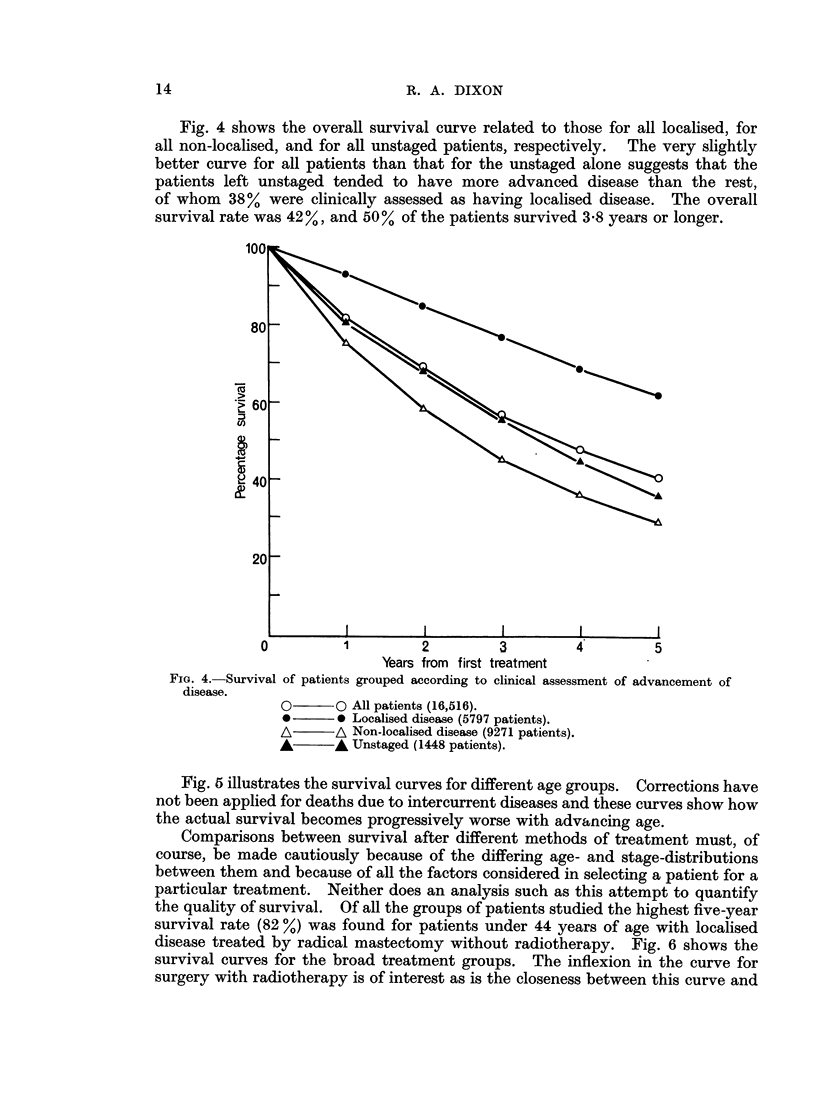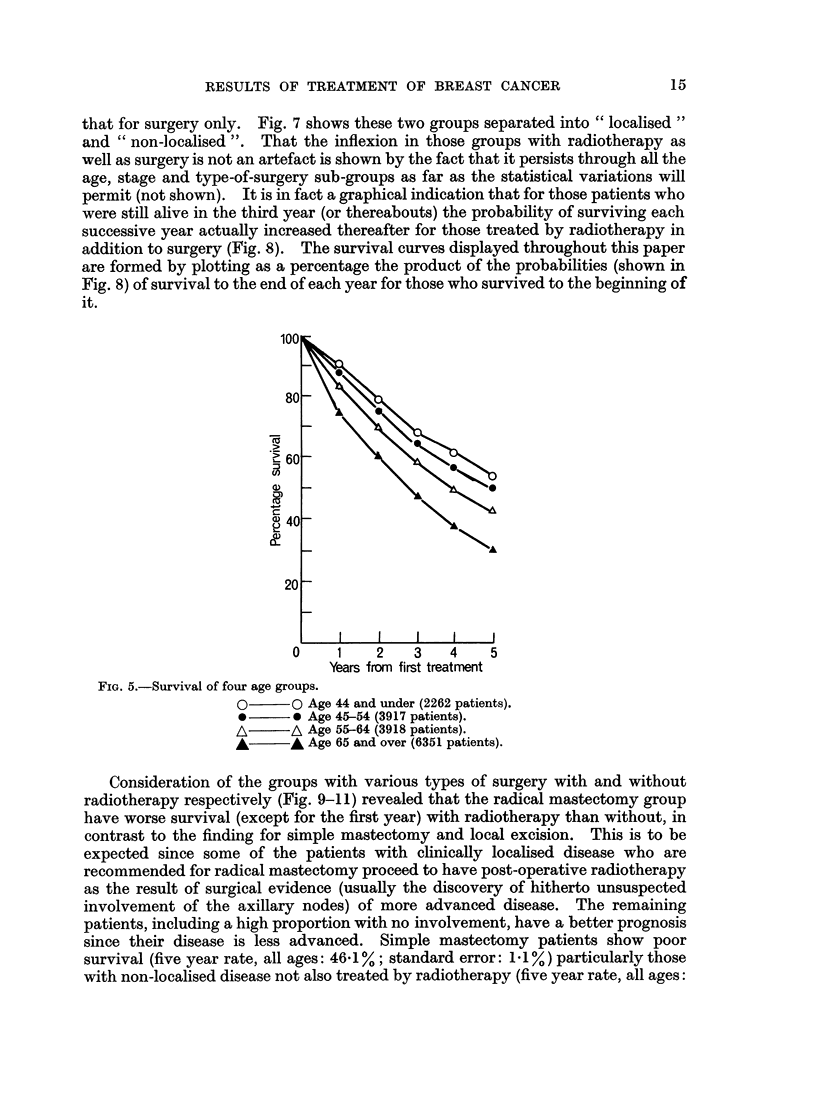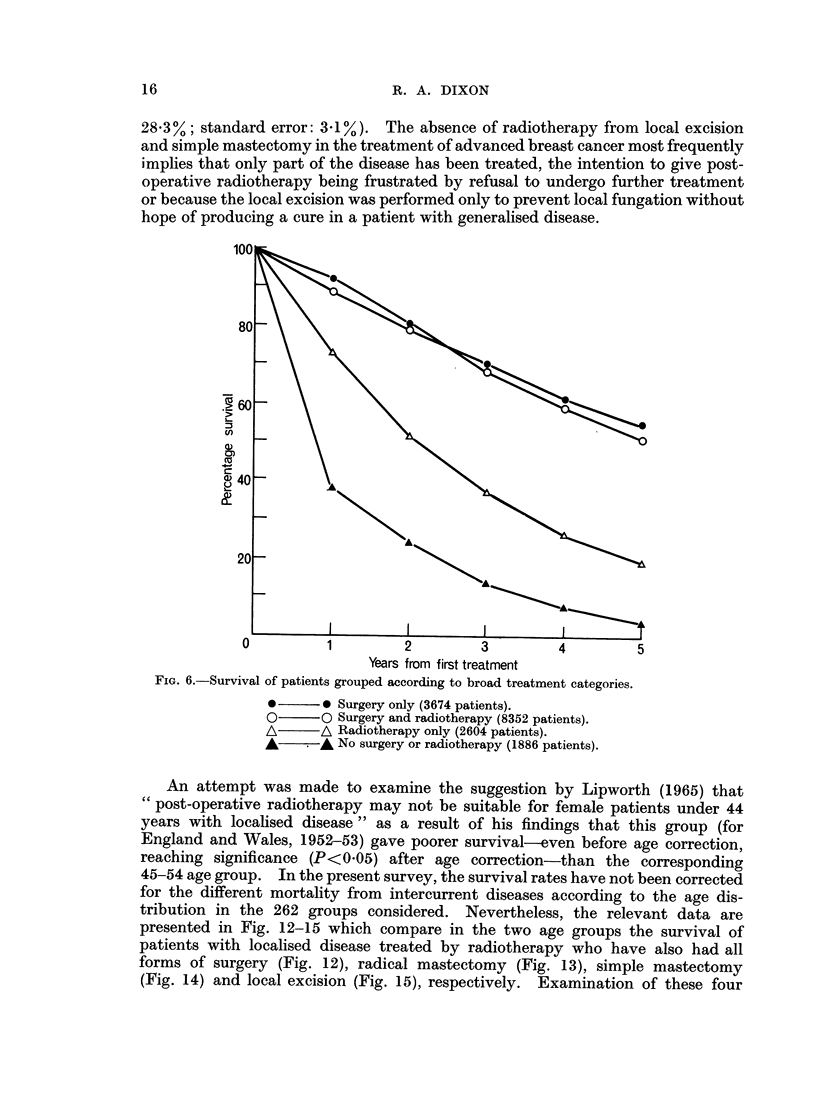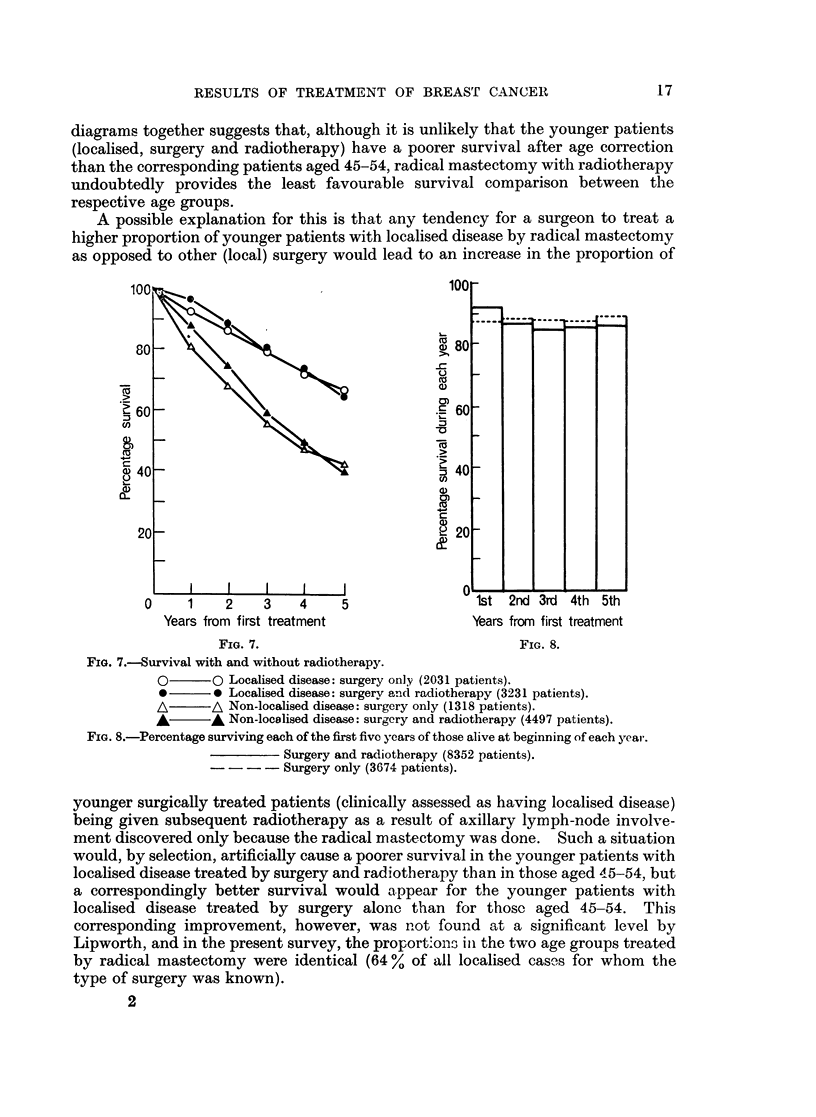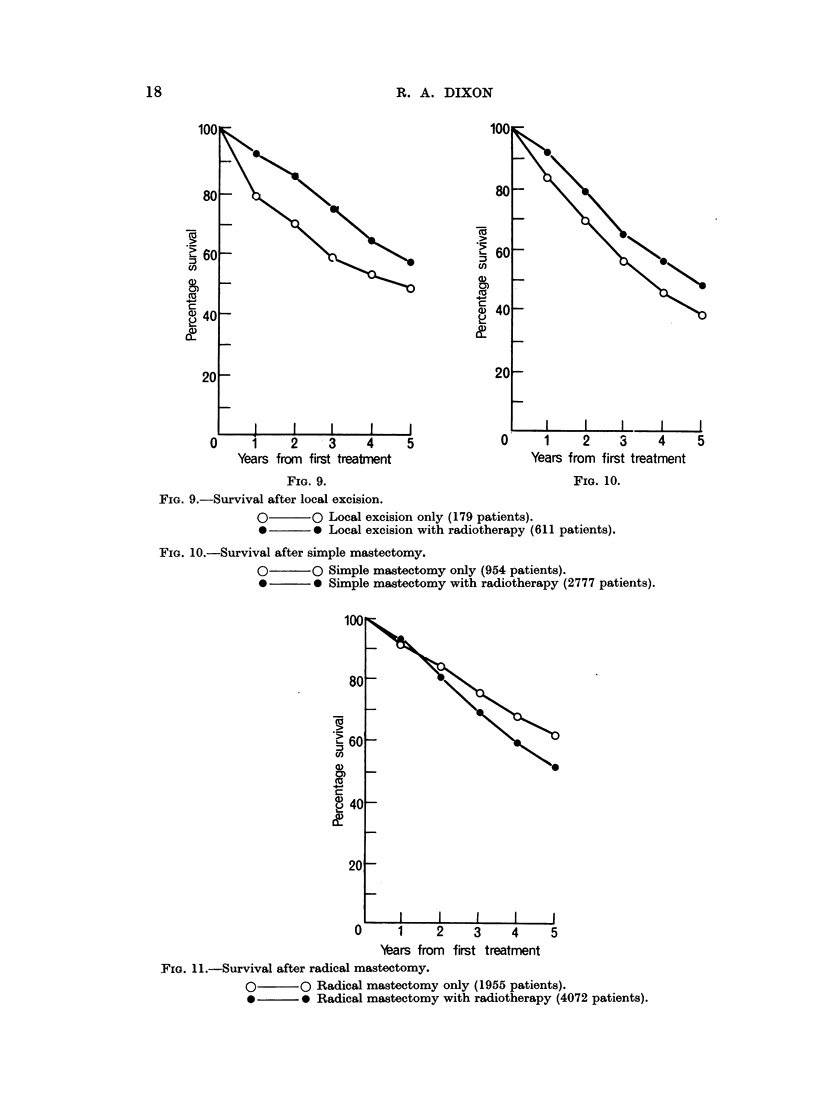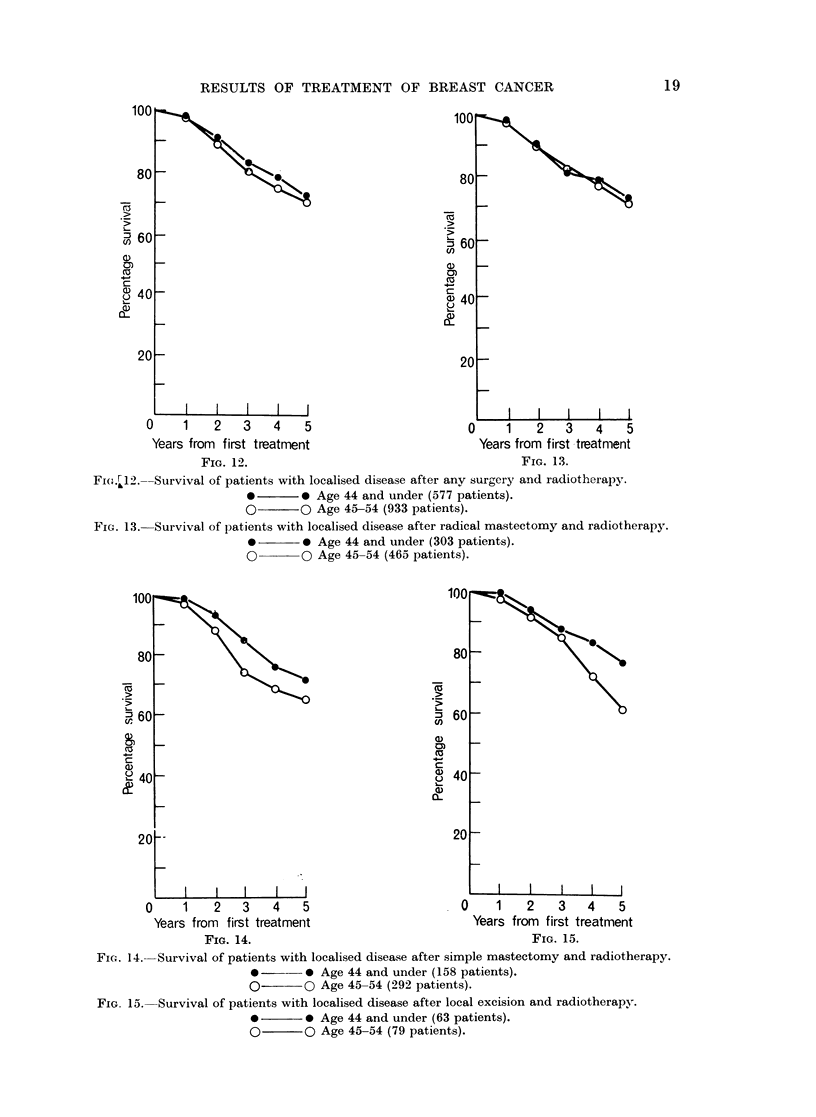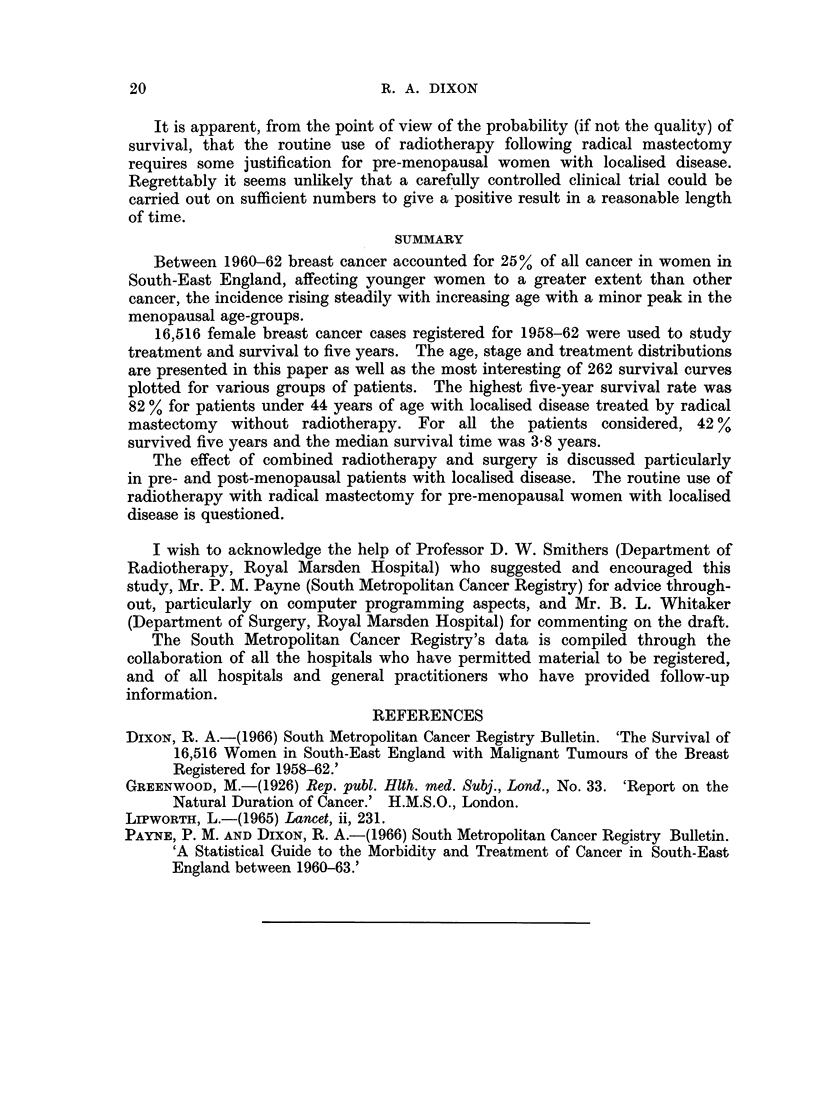# Results of treatment of breast cancer in South-East England.

**DOI:** 10.1038/bjc.1969.2

**Published:** 1969-03

**Authors:** R. A. Dixon


### RESULTS OF TREATMENT OF BREAST CANCER IN SOUTH-EAST ENGLAND

R. A. DIXON*

*From the South Metropolitan Cancer Registry, Sutton, Surrey*

THE results of treatment of groups of patients with primary malignant breast tumours treated by individuals in single hospitals are published from time to time. These all represent selection from the population with the disease and most of them are published in order to support one treatment method or some combination of treatment methods adopted by the person or institution involved. Controlled clinical trials are overcoming many of the difficulties of comparison created by these reports which must almost inevitably have their under-currents of local pride. This report attempts to look at a fairly well defined population and to see what happens to patients with breast cancer treated by many different people using different combinations of treatment in different hospitals. No local pride is involved; no treatment method is being advocated.

### *Morbidity of Breast Cancer*

Between 1960 and 1962, during which regional registration of malignant disease at the South Metropolitan Cancer Registry first became very nearly complete, an annual average of 3247 cases of breast cancer was reported among women actually resident within the S.M.C.R. region. This corresponds to an annual registration rate of 7·4 per 10,000 of the population, accounting for 25% of female cancer. The annual age-specific registration rates of all cancer for each sex, and of female breast cancer, during this period are shown in Fig. 1 (Payne and Dixon, 1966). The influence of the menopause on the morbidity of breast cancer is apparent. The age distributions of all cancer for each sex (Fig. 2) (Payne and Dixon, 1966) illustrate the effect of high breast cancer morbidity among women under 50 on the age distribution of all female cancer during the period 1960–62.

### *The Series used to Determine Treatment and Survival*

By the end of 1965, when this study began, 16,516 cases of primary breast tumours in women, first diagnosed during the period 1958–62, had been reported to the South Metropolitan Cancer Registry. Most of the patients were resident in the Wessex, the South West Metropolitan or the South East Metropolitan Hospital Boards' areas, although some (approximately 1200), attending hospitals within this region, lived elsewhere. Since this study is concerned primarily with survival rates up to five years after first treatment, and not with morbidity within the region, patients resident outside the region were not excluded.

It was decided to group the 16,516 cases according to the clinical extent of the disease, surgical treatment, radiotherapy, and age. (More than 20% of the

*Present address: Department of Preventive Medicine and Public Health and Department of Applied Mathematics and Computing Science, The University, Sheffield 10.

FIG. 1.—Annual age-specific registration rates. (South Metropolitan Cancer Registry, 1960–62).

●——● All male cancer.

○——○ All female cancer.

×——× Female breast cancer.

FIG. 2.—Age distribution of all cancers (South Metropolitan Cancer Registry, 1960–62)

●——● Males. ○——○ Females.

patients underwent endocrine surgery or irradiation or received hormones or chemotherapy but none of these factors was used to classify the patients.) Regrettably, clinical staging of breast cancer at the hospitals where the patients were first seen was not sufficiently widespread to permit more precise classification than into " localised " and " non-localised " disease in a survey such as this. Indeed, a proportion (8·8%) had to be left completely unstaged. The criteria used for separation into " localised " and " non-localised " were those adopted by Lipworth (1965) and relate to detectable spread assessed by clinical examination of the patients, in some cases assisted by radiological investigations. Those with no spread beyond the breast tissue, no involvement of the axillary nodes, and no metastases were considered to have localised disease. Skin involvement in direct continuity with the tumour and not wide of it did not preclude the growth from being considered as localised.

In terms of the well-known TNM classification recommended by the International Union Against Cancer, non-localised disease was assumed to be present if any one or more of the following condititions was satisfied:

Primary tumour = T4
Regional lymph nodes = N1, N2 or N3
Distant metastases = M1

Where clinical considerations indicated that none of these was satisfied, localised disease was assumed. The tumour was designated " unstaged " where insufficient information was recorded in the hospital case-notes. Methods of reducing the number which could not be staged became apparent. For example, the few patients upon whom radical mastectomies were performed and yet whose treatments were described before-hand as palliative undoubtedly had advanced disease.

*Treatment*

Fig. 3 shows how the broad treatment combinations were used in various age groups, and Table I shows the use made of different surgery. 50·6% of all the

TABLE I.—*Type of Surgery Used in Various Age Groups: South Metropolitan Cancer Registry* (1958–62)

| Age group | Without surgery | With surgery: Local excision | Simple mastectomy | Radical mastectomy | Unspecified surgery | All surgery | Totals with or without surgery |
|---|---|---|---|---|---|---|---|
| Age unknown | 22 | 1 | 10 | 28 | 7 | 46 | 68 |
| Under 44 | 349 | 152 | 424 | 1127 | 210 | 1913 | 2262 |
| 45–54 | 730 | 176 | 761 | 1853 | 397 | 3187 | 3917 |
| 55–64 | 945 | 173 | 856 | 1584 | 360 | 2973 | 3918 |
| Over 65 | 2444 | 288 | 1680 | 1435 | 504 | 3907 | 6351 |
| All ages | 4490 | 790 | 3731 | 6027 | 1478 | 12026 | 16516 |

patients were treated by surgery and radiotherapy, and only 22·2% by surgery alone. This is quite different from Lipworth's findings (1965) that " in some regions of England and Wales surgery is at present used more frequently in the treatment of breast cancer than surgery combined with radiation ". It has been shown (Dixon, 1966) that this was not due to an influx of patients from other areas in which radiotherapy facilities were less readily available. Those who are accustomed to hospital series may find the percentage of patients untreated by surgery or radiotherapy (11%) rather high. However, this largely indicates the percentage of patients registered eventually (perhaps from a death certificate) as having had breast cancer, but who did not attend hospital.

FIG. 3.—Broad types of treatment used in various age groups (South Metropolitan Cancer Registry, 1958–62).

The term " unspecified surgery " applies to those patients for whom there was not sufficient information to say whether the surgery given was local excision, simple mastectomy or radical mastectomy. It refers only to treatment of the primary tumour and does not include oophorectomy, adrenalectomy, hypophysectomy or removal of metastases. Although there is no intrinsic interest in the survival rate of this category or indeed of those for whom the age or stage were not known, they are nevertheless retained to contribute information to the various sub-totals (e.g. over all ages) and to the grand total of all breast cancer patients.

*Survival*

Table I can be sub-divided according to whether or not radiotherapy was given, and then according to the clinical stage categories " localised ", " non-localised " or " unstaged ". Ultimately 504 different groupings of patients can be made in this way for analysis of survival characteristics. Life-tables† (Greenwood, 1926) were constructed from these groupings and showed for each of the first five years the numbers alive, dead and untraced, the interval survival rate within that year alone and the cumulative survival rate up to and including that year (and the associated standard error). The cumulative one-, three-, and five-year survival rates (with their standard errors) for some of the more interesting larger groups of patients are given in Table II.

TABLE II.—*Summary of Survival Rates for Some Large Groups of Patients*

| Group | Number of cases | Survival rates (with standard errors in parentheses) 1 year | 3 years | 5 years |
|---|---|---|---|---|
| All patients | 16516 | 82·0 (0·3) | 57·5 (0·4) | 41·7 (0·5) |
| Localised disease | 5797 | 93·3 (0·3) | 76·8 (0·6) | 69·1 (0·7) |
| Non-localised disease | 9271 | 75·3 (0·5) | 45·9 (0·8) | 30·0 (0·6) |
| Treated by surgery alone | 3674 | 88·3 (0·5) | 69·9 (0·8) | 55·5 (1·1) |
| Treated by radiotherapy alone | 2604 | 72·6 (0·9) | 37·3 (1·0) | 20·1 (1·0) |
| Treated by surgery and radiotherapy | 8352 | 92·2 (0·3) | 68·6 (0·6) | 51·9 (0·7) |
| Treated by surgery with or without radiotherapy | 12026 | 91·1 (0·3) | 69·0 (0·5) | 53·0 (0·6) |
| Not treated by surgery or radiotherapy | 1886 | 38·2 (1·1) | 14·0 (0·8) | 4·7 (0·6) |
| Local excision with or without radiotherapy | 790 | 89·5 (1·1) | 71·2 (1·8) | 55·5 (2·5) |
| Simple mastectomy with or without radiotherapy | 3731 | 89·7 (0·5) | 63·2 (0·9) | 46·1 (1·1) |
| Radical mastectomy with or without radiotherapy | 6027 | 92·6 (0·3) | 72·1 (0·6) | 55·4 (0·9) |
| Under 44 | 2262 | 90·7 (0·6) | 69·0 (1·1) | 55·2 (1·4) |
| 45–54 | 3917 | 87·8 (0·5) | 65·2 (0·8) | 51·0 (1·0) |
| 55–64 | 3918 | 83·6 (0·6) | 58·9 (0·8) | 42·9 (1·0) |
| Over 65 | 6351 | 74·3 (0·6) | 47·5 (0·7) | 30·4 (0·8) |

† This method uses information contributed by all patients up to the time when they become " untraced ", rather than excluding completely those who are lost sight of at any stage, as is often done to simplify the calculations. (A computer programme was written to construct the life-tables from 15 parameters for each of 262 selected groups out of the 504 possible groups, and to calculate the survival rates with their standard errors.) For this reason comparisons of survival rates should be made only within this paper and not with crude rates given elsewhere. However the rates given here are not age-standardised for mortality from intercurrent diseases, although many are quoted for specific age groups. Indeed these may be of more practical interest since they represent the actual percentage of patients in a particular age-group who can be expected to survive the stated period, and therefore provide a realistic measure which can be considered in assessing the value of major surgery.

Fig. 4 shows the overall survival curve related to those for all localised, for all non-localised, and for all unstaged patients, respectively. The very slightly better curve for all patients than that for the unstaged alone suggests that the patients left unstaged tended to have more advanced disease than the rest, of whom 38% were clinically assessed as having localised disease. The overall survival rate was 42%, and 50% of the patients survived 3·8 years or longer.

FIG. 4.—Survival of patients grouped according to clinical assessment of advancement of disease.

○——○ All patients (16,516).
●——● Localised disease (5797 patients).
△——△ Non-localised disease (9271 patients).
▲——▲ Unstaged (1448 patients).

Fig. 5 illustrates the survival curves for different age groups. Corrections have not been applied for deaths due to intercurrent diseases and these curves show how the actual survival becomes progressively worse with advancing age.

Comparisons between survival after different methods of treatment must, of course, be made cautiously because of the differing age- and stage-distributions between them and because of all the factors considered in selecting a patient for a particular treatment. Neither does an analysis such as this attempt to quantify the quality of survival. Of all the groups of patients studied the highest five-year survival rate (82%) was found for patients under 44 years of age with localised disease treated by radical mastectomy without radiotherapy. Fig. 6 shows the survival curves for the broad treatment groups. The inflexion in the curve for surgery with radiotherapy is of interest as is the closeness between this curve and that for surgery only. Fig. 7 shows these two groups separated into " localised " and " non-localised ". That the inflexion in those groups with radiotherapy as well as surgery is not an artefact is shown by the fact that it persists through all the age, stage and type-of-surgery sub-groups as far as the statistical variations will permit (not shown). It is in fact a graphical indication that for those patients who were still alive in the third year (or thereabouts) the probability of surviving each successive year actually increased thereafter for those treated by radiotherapy in addition to surgery (Fig. 8). The survival curves displayed throughout this paper are formed by plotting as a percentage the product of the probabilities (shown in Fig. 8) of survival to the end of each year for those who survived to the beginning of it.

FIG. 5.—Survival of four age groups.

○——○ Age 44 and under (2262 patients).
●——● Age 45–54 (3917 patients).
△——△ Age 55–64 (3918 patients).
▲——▲ Age 65 and over (6351 patients).

Consideration of the groups with various types of surgery with and without radiotherapy respectively (Fig. 9–11) revealed that the radical mastectomy group have worse survival (except for the first year) with radiotherapy than without, in contrast to the finding for simple mastectomy and local excision. This is to be expected since some of the patients with clinically localised disease who are recommended for radical mastectomy proceed to have post-operative radiotherapy as the result of surgical evidence (usually the discovery of hitherto unsuspected involvement of the axillary nodes) of more advanced disease. The remaining patients, including a high proportion with no involvement, have a better prognosis since their disease is less advanced. Simple mastectomy patients show poor survival (five year rate, all ages: 46·1%; standard error: 1·1%) particularly those with non-localised disease not also treated by radiotherapy (five year rate, all ages:

28·3%; standard error: 3·1%). The absence of radiotherapy from local excision and simple mastectomy in the treatment of advanced breast cancer most frequently implies that only part of the disease has been treated, the intention to give post-operative radiotherapy being frustrated by refusal to undergo further treatment or because the local excision was performed only to prevent local fungation without hope of producing a cure in a patient with generalised disease.

FIG. 6.—Survival of patients grouped according to broad treatment categories.

●———● Surgery only (3674 patients).
○———○ Surgery and radiotherapy (8352 patients).
△———△ Radiotherapy only (2604 patients).
▲———▲ No surgery or radiotherapy (1886 patients).

An attempt was made to examine the suggestion by Lipworth (1965) that "post-operative radiotherapy may not be suitable for female patients under 44 years with localised disease" as a result of his findings that this group (for England and Wales, 1952–53) gave poorer survival—even before age correction, reaching significance ($P<0{\cdot}05$) after age correction—than the corresponding 45–54 age group. In the present survey, the survival rates have not been corrected for the different mortality from intercurrent diseases according to the age distribution in the 262 groups considered. Nevertheless, the relevant data are presented in Fig. 12–15 which compare in the two age groups the survival of patients with localised disease treated by radiotherapy who have also had all forms of surgery (Fig. 12), radical mastectomy (Fig. 13), simple mastectomy (Fig. 14) and local excision (Fig. 15), respectively. Examination of these four diagrams together suggests that, although it is unlikely that the younger patients (localised, surgery and radiotherapy) have a poorer survival after age correction than the corresponding patients aged 45–54, radical mastectomy with radiotherapy undoubtedly provides the least favourable survival comparison between the respective age groups.

A possible explanation for this is that any tendency for a surgeon to treat a higher proportion of younger patients with localised disease by radical mastectomy as opposed to other (local) surgery would lead to an increase in the proportion of

FIG. 7.

FIG. 8.

FIG. 7.—Survival with and without radiotherapy.

○——○ Localised disease: surgery only (2031 patients).
●——● Localised disease: surgery and radiotherapy (3231 patients).
△——△ Non-localised disease: surgery only (1318 patients).
▲——▲ Non-localised disease: surgery and radiotherapy (4497 patients).

FIG. 8.—Percentage surviving each of the first five years of those alive at beginning of each year.

———— Surgery and radiotherapy (8352 patients).
— — — — Surgery only (3674 patients).

younger surgically treated patients (clinically assessed as having localised disease) being given subsequent radiotherapy as a result of axillary lymph-node involvement discovered only because the radical mastectomy was done. Such a situation would, by selection, artificially cause a poorer survival in the younger patients with localised disease treated by surgery and radiotherapy than in those aged 45–54, but a correspondingly better survival would appear for the younger patients with localised disease treated by surgery alone than for those aged 45–54. This corresponding improvement, however, was not found at a significant level by Lipworth, and in the present survey, the proportions in the two age groups treated by radical mastectomy were identical (64 % of all localised cases for whom the type of surgery was known).

Fig. 9.

Fig. 10.

Fig. 9.—Survival after local excision.

○———○ Local excision only (179 patients).
●———● Local excision with radiotherapy (611 patients).

Fig. 10.—Survival after simple mastectomy.

○———○ Simple mastectomy only (954 patients).
●———● Simple mastectomy with radiotherapy (2777 patients).

Fig. 11.—Survival after radical mastectomy.

○———○ Radical mastectomy only (1955 patients).
●———● Radical mastectomy with radiotherapy (4072 patients).

Fig. 12.

Fig. 13.

Fig. 12.—Survival of patients with localised disease after any surgery and radiotherapy.

● —— ● Age 44 and under (577 patients).

○ —— ○ Age 45–54 (933 patients).

Fig. 13.—Survival of patients with localised disease after radical mastectomy and radiotherapy.

● —— ● Age 44 and under (303 patients).

○ —— ○ Age 45–54 (465 patients).

Fig. 14.

Fig. 15.

Fig. 14.—Survival of patients with localised disease after simple mastectomy and radiotherapy.

● —— ● Age 44 and under (158 patients).

○ —— ○ Age 45–54 (292 patients).

Fig. 15.—Survival of patients with localised disease after local excision and radiotherapy.

● —— ● Age 44 and under (63 patients).

○ —— ○ Age 45–54 (79 patients).

It is apparent, from the point of view of the probability (if not the quality) of survival, that the routine use of radiotherapy following radical mastectomy requires some justification for pre-menopausal women with localised disease. Regrettably it seems unlikely that a carefully controlled clinical trial could be carried out on sufficient numbers to give a positive result in a reasonable length of time.

SUMMARY

Between 1960–62 breast cancer accounted for 25% of all cancer in women in South-East England, affecting younger women to a greater extent than other cancer, the incidence rising steadily with increasing age with a minor peak in the menopausal age-groups.

16,516 female breast cancer cases registered for 1958–62 were used to study treatment and survival to five years. The age, stage and treatment distributions are presented in this paper as well as the most interesting of 262 survival curves plotted for various groups of patients. The highest five-year survival rate was 82% for patients under 44 years of age with localised disease treated by radical mastectomy without radiotherapy. For all the patients considered, 42% survived five years and the median survival time was 3·8 years.

The effect of combined radiotherapy and surgery is discussed particularly in pre- and post-menopausal patients with localised disease. The routine use of radiotherapy with radical mastectomy for pre-menopausal women with localised disease is questioned.

I wish to acknowledge the help of Professor D. W. Smithers (Department of Radiotherapy, Royal Marsden Hospital) who suggested and encouraged this study, Mr. P. M. Payne (South Metropolitan Cancer Registry) for advice throughout, particularly on computer programming aspects, and Mr. B. L. Whitaker (Department of Surgery, Royal Marsden Hospital) for commenting on the draft.

The South Metropolitan Cancer Registry's data is compiled through the collaboration of all the hospitals who have permitted material to be registered, and of all hospitals and general practitioners who have provided follow-up information.

### REFERENCES

DIXON, R. A.—(1966) South Metropolitan Cancer Registry Bulletin. 'The Survival of 16,516 Women in South-East England with Malignant Tumours of the Breast Registered for 1958–62.'

GREENWOOD, M.—(1926) *Rep. publ. Hlth. med. Subj., Lond.*, No. 33. 'Report on the Natural Duration of Cancer.' H.M.S.O., London.

LIPWORTH, L.—(1965) *Lancet*, ii, 231.

PAYNE, P. M. AND DIXON, R. A.—(1966) South Metropolitan Cancer Registry Bulletin. 'A Statistical Guide to the Morbidity and Treatment of Cancer in South-East England between 1960–63.'