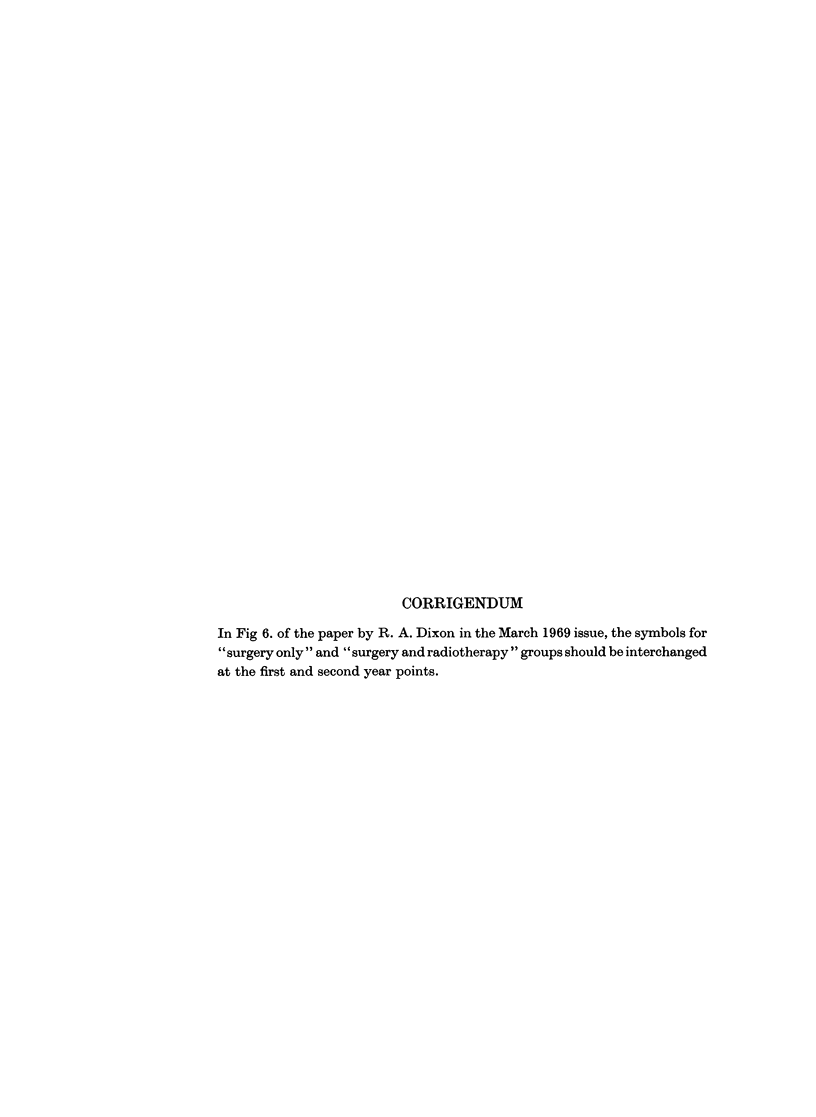# Corrigendum

**Published:** 1969-03

**Authors:** 


### CORRIGENDUM

In Fig 6. of the paper by R. A. Dixon in the March 1969 issue, the symbols for "surgery only" and "surgery and radiotherapy" groups should be interchanged at the first and second year points.